# Banning highly hazardous pesticides saves the lives of young people, particularly females, in low- and middle-income countries

**DOI:** 10.1186/s12889-023-17071-y

**Published:** 2023-11-15

**Authors:** Lisa Schölin, Duleeka Knipe, Piumee Bandara, Michael Eddleston, Aastha Sethi

**Affiliations:** 1 Centre for Pesticide Suicide Prevention, University of Edinburgh, Edinburgh, UK; 2 Bristol Medical School, Population Health Sciences, University of Bristol, Bristol, UK; 3 South Asian Clinical Toxicology Research Collaboration, Faculty of Medicine, University of Peradeniya, Peradeniya, Sri Lanka

**Keywords:** Suicide, Self-poisoning, Pesticide, Bans, Prevention

## Abstract

Pesticide self-poisoning is a public health problem mostly affecting low- and middle-income countries. In Sri Lanka, India and China suicide rates have reduced among young people, particularly females, following highly hazardous pesticides (HHP) bans. This success story requires attention to encourage more research on differential effects of HHP bans.

## Introduction

Suicide is a major public health issue. More than 700,000 people die by suicide each year and it ranks as the fourth leading cause of death among young people aged 15–29 years [1]. Self-poisoning with pesticides (particularly insecticides, herbicides, and fumigants) [2] is one of the most common means of suicide, accounting for 14–20% of suicides globally [3]. The majority of these deaths occur in low- and middle-income countries (LMICs), predominantly in Asia, although it is notable that data for LMICs in the African and Eastern Mediterranean Region are lacking [3]. In LMICs, pesticides of high acute toxicity, known as highly hazardous pesticides (HHPs), are often readily available in moments of crisis, resulting in many deaths ('suicides') in people who did not intend to die and where the self-poisoning event had little premeditation [4–6].

Among pesticides used across the world, HHPs constitute a small proportion of the total amount used, but their impact on health and environment is disproportionately detrimental [7]. To address this issue, international agencies (in particular the Food and Agriculture Organization of the United Nations and World Health Organization [WHO]) have developed guidelines and policies to support countries in strengthening pesticide regulation [1, 8, 9]. However, attention to the problems of HHP poisoning started in the mid-2000s with a WHO-led global campaign that particularly focused on safe storage [10]. More recently, in May 2023, the World Health Assembly approved a landmark resolution calling on WHO and its Member States to scale up efforts to reduce the impact of chemicals (including HHPs), waste, and pollution on human health [11]. Despite these efforts, pesticide sales have grown greatly, with escalation of marketing in LMICs where regulatory systems are weak, undermining global efforts to phase out HHPs [12]. This raises concerns and questions about commercial actors’ involvement in suicide prevention, which has been discussed in recent work elsewhere [13].

## Access to HHPs and sex differences in suicide rates

Contrary to high-income countries (HICs), the male-to-female suicide ratio tends to be lower in LMICs and the highest female suicide rates globally are found in the WHO South-East Asia region [14]. When breaking this down by age, data shows that for example in India, the suicide rate is higher among females than males in all age groups under 25 years [15]. Data from Sri Lanka shows that, in 2010–12, among females the highest suicide rate was in the age group 17–21 years, in contrast to males where the highest rate was among those aged ≥ 60 years [16]. In countries where pesticides are available, it may be therefore important to look in more detail into differences in suicide rates by sex and age.

One major contributing factor to these sex and age differences in suicide rates in LMICs is access to HHPs. Self-poisoning is a common method of self-harm in young females globally [17, 18]. Given the limited access to pesticides, self-poisoning among young females in HICs has low case fatality and results in relatively few deaths (‘suicides’) relative to the number of self-harm events. Many of these young females are given a second chance and the opportunity to live healthy lives – many do not self-harm again [19, 20]. In contrast, in LMICs, HHPs are easily available from local shops and within households, particularly in rural communities [18, 21, 22]. As a result, acts of pesticide self-poisoning that are typically carried out in moments of crisis, without any suicidal intent, resulting in death [6].

Despite their well-documented association with suicide and other adverse health consequences in addition to the negative impacts on the environment, HHPs continue to be manufactured and exported to LMICs [23]. In most cases, countries that are manufacturing HHPs have deemed these products too unsafe for their own population but continue to export and promote sales to countries that do not have the regulatory systems in place to restrict their import and use [23]. This represents a major injustice and inequity regarding government restrictions on supply and use of substances known to be poisonous. Agriculture practices also differ across the world. In LMICs, many small holder farmers have direct access to high concentration pesticide formulations, in contrast to HICs where only a small number of individuals work on the land and have access to pesticides [24]. However, regulation of HHPs to remove them from agriculture, and therefore from rural households, can reduce suicide rates without negative impacts on agricultural yield [25, 26].

## Declining suicide rates are particularly prominent among young females

Research in three countries that have implemented regulations over time (China, Sri Lanka and India), has shown that the greatest proportional changes in suicide rates occurred among young females. These three countries, of which China and India constitute a high proportion of the world’s suicides [14], are among the few countries that have sex- and age-disaggregated data available over time which demonstrate these changes by sex and age.

**Fig. 1:**
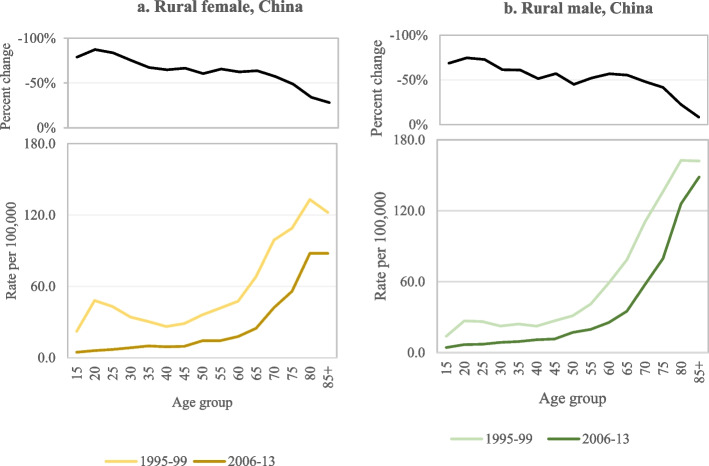
**a**-**b** Suicide rate per 100,000 and percent change in suicide rate in rural areas by sex and age in China, 1995–99 [28] and 2006–12 [29]

In China, where an estimated 62% of suicides in 1995–99 were due to pesticide self-poisoning [27], there was a peak among young rural females [28]. By 2006–13, following bans of acutely toxic HHPs and extensive migration to cities, suicide rates had drastically reduced with a remarkable reduction in deaths among young females in rural areas. Among rural males, this change was not as drastic even though a reduction was evident also among younger rural males (Fig. 1a, b) [29].

**Fig. 2:**
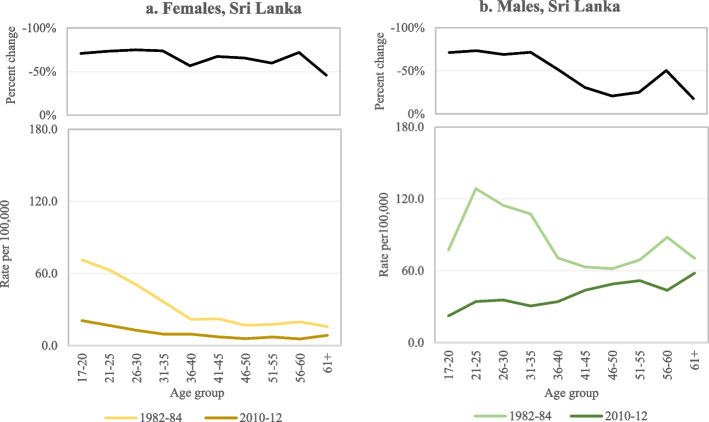
**a-b** Suicide rate and percent change in suicide rate by sex and age in Sri Lanka, 1982–82 and 2010–12 [16]

**Fig. 3:**
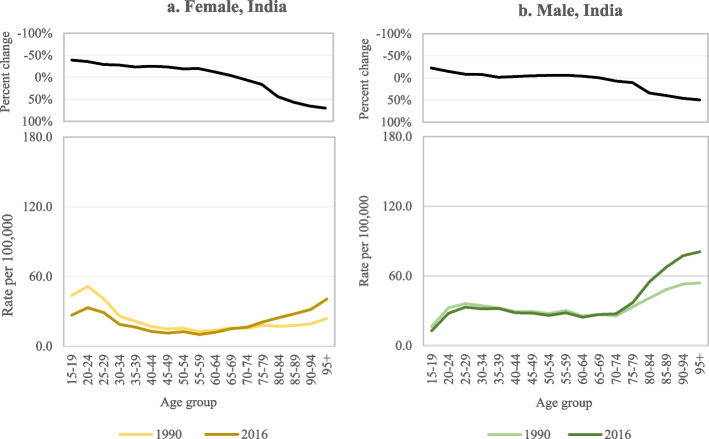
**a-b** Suicide rate and percent change in suicide rate by sex and age in India, 1990 and 2016 [15]

In Sri Lanka, where around 80% of all suicides were due to pesticide self-poisoning in the mid-1990s [30], HHP bans over several decades resulted in dramatic reductions in suicide rates [31]. Here, particularly marked reductions in suicide rate were evident in the youngest age groups among both males and females (Fig. 2a, b), though females in all age groups had greater reductions in suicide rates than males [16]. In India, 19% of all suicide cases in 2001 were due to ‘insecticide’ self-poisoning [32]. There was a decline in age-specific standardised suicide rate between 1990 and 2016, which was greater among young females than males [15]. The percent reductions in suicide rate in females and males, respectively, were 39% and 22% among those aged 15–19 years, 36% and 15% in those aged 20–24 years, and 29% and 9% in those aged 25–29 years (Fig. 3a, b) following bans on several HHPs [15]. Of note, however, there is still a large excess of deaths in young females in India and increased suicide rates in the oldest age groups over the same period in both males and females [15]. Fortunately, the government is currently considering additional bans of HHPs responsible for many self-poisoning deaths [33].

## Data limitations

It should be noted that sex-disaggregated data on pesticide suicide, in the context of changes to regulating access to HHPs, are limited. More detailed data on sex- and method-specific suicide rates would allow for stronger conclusions of reductions in pesticide self-poisonings in contrast to other types of self-poisoning, however these types of studies are currently lacking. Research on sex-disaggregated data over time however suggests differential effects based on suicide methods. A study using Indian data, following a ban on endosulfan in 2011, showed that the overall female suicide rate declined as deaths from pesticide self-poisoning fell, with only a modest increase in use of other methods; however, in males an increase in hanging largely offset the decrease in pesticide suicides [34]. This may however have varied across age groups.

Further research on the impact of pesticide bans in countries where pesticide suicide accounts for a high proportion of suicide deaths, by sex and age, is needed as well as explorations of potential reasons for differential impact by sex and age, within different countries. This will require availability of disaggregated data that can aid research and intervention development for suicide and self-harm prevention in LMICs where a high number of young females are still dying by pesticide suicide [35, 36]. Furthermore, India records suicide for the categories male, female and transgender, however Indian research studies tend to only record male and female [35] which is also the case for studies from other countries. This is an area that requires attention globally in future research as binary categorisation might hide issues with elevated suicide rates in those who don’t identify as male/female.

## HHP bans are effective interventions but not a panacea

HHP bans are only one part of suicide prevention and it has been pointed out, for example in India, that “targeting of interventions is needed by age, sex, and other factors” (p. 162) due to the number of underlying factors involved in suicide deaths [37]. Yet, the available data suggests that bans may be an intervention that specifically impacts on the suicide rate among young females. Therefore, the effect of HHP bans on reducing suicide in young people, especially young females, is important. However, self-harm is often associated with feelings of disempowerment [38, 39]. The context in which females live and how their lives are restricted often leads to high levels of distress, which may be compounded in the context of gender inequality, poverty, domestic violence, and alcohol misuse [38, 39]. This often leads to higher rates of self-harm and suicide among females in LMICs [35–41], particularly in young ages [16]. A public health approach to suicide prevention through a socioecological and life course approach offers broader conceptual models that take into consideration the multifaceted and complex nature of suicide [42].

## Conclusions

Whilst the banning of HHPs is not going to address the deep-rooted socio-cultural determinants of suicide, which may take time to change, it affords us time to help those in distress and ultimately save lives. A WHO cost-effectiveness study indicated that national bans on HHPs can be a cost-effective intervention for reducing suicide deaths in countries with a high burden of suicides attributable to pesticides [43]. This does not require banning all or even many pesticides. Among pesticides used across the world, HHP constitutes a small proportion of the total use but their impact on health and environment is disproportionally detrimental [7]. Too many young people in LMICs have easy access to acutely toxic HHPs at times of distress, and the stark differences in exposure to HHPs across the world highlights major inequities in global health. National bans on HHPs will save the lives of many young females that would otherwise have ended far too soon. Restricting access to means is the most effective strategy for suicide prevention because many acts of self-harm are impulsive and ambivalent in intent [41]. Many pesticide suicides can be prevented when acutely toxic HHPs are replaced with pesticides of low toxicity [1, 44, 45] and when self-harm events do occur, they are less likely to be fatal.

## Data Availability

Not applicable.

## Abbreviations

HHPs: Highly hazardous pesticides
LMICs: Low- and middle-income countries

## References

[1] WHO and FAO. *Preventing suicide: a resource for pesticide registrars and regulators.* World Health Organization, Geneva, 2019. Available from: https://www.who.int/publications/i/item/9789241516389. Accessed 24 Jan 2023.

[2] WHO. *Preventing suicide: A global imperative,* World Health Organization. Geneva. 2014. Available from: https://www.who.int/publications/i/item/9789241564779. Accessed 24 Jan 2023.

[3] Mew EJ, Padmanathan P, Konradsen F, Eddleston M, Chang S, Phillips MR (2017). The global burden of fatal self-poisoning with pesticides 2006–15: systematic review. J Affect Disord.

[4] Eddleston M, Karunaratne A, Weerakoon M, Kumarasinghe S, Rajapakshe M, Sheriff RMH (2006). Choice of poison for intentional self-poisoning in rural Sri Lanka. Clin Toxicol (Phila).

[5] Conner KR, Phillips MR, Meldrum S, Knox KL, Zhang Y, Yang G (2005). Low-planned suicides in China. Psychol Med.

[6] Pearson V, Phillips MR, He F, Ji H (2002). Attempted suicide among young rural women in the People’s Republic of China: possibilities for prevention. Suicide Life Threat Behav.

[7] FAO and WHO. *Detoxifying agriculture and health from highly hazardous pesticides - A call for action*. Food and Agriculture Organzation, Rome. 2019. Available at: https://apps.who.int/iris/bitstream/handle/10665/330659/9789241517065-eng.pdf. Accessed 24 Jan 2023.

[8] WHO and FAO. *The International Code of Conduct on Pesticide Management*. Food and Agriculture Organzation, Rome. 2014. Available at: https://www.who.int/publications/i/item/9789251085493. Accessed 15 Aug 2023.

[9] WHO. LIVE LIFE: *An implementation guide for suicide prevention in countries*. World Health Organization, Geneva. 2021. Available at: https://www.who.int/publications/i/item/9789240026629. Accessed 15 Aug 2023.

[10] WHO. Safer access to pesticides: community intervetions. World Health Organization, Geneva. 2006. Available at: https://apps.who.int/iris/handle/10665/43585. Accessed 15 Aug 2023.

[11] WHO. Seventy-sixth World Health Assembly, 21–30 May 2023. The impact of chemicals, waste and pollution on human health, A76/ A/CONF./2. World Health Organization, Geneva. Available at: https://apps.who.int/gb/ebwha/pdf_files/WHA76/A76_ACONF2-en.pdf. Accessed 15 Aug 2023.

[12] UNEP. *Synthesis Report on the Environmental and Health Impacts of Pesticides and Fertilizers and Ways to Minimize Them*. United Nations Environment Programme, Geneva. 2022. Available at: https://www.unep.org/resources/report/environmental-and-health-impacts-pesticides-and-fertilizers-and-ways-minimizing. Accessed 15 Aug 2023.

[13] van Schalkwyk MCI, Collin J, Eddleston M, Petticrew M, Pearson M, Schölin L (2023). Conceptualising the commercial determinants of suicide: broadening the lens on suicide and self-harm prevention. Lancet Psychiatry.

[14] WHO. *Suicide worldwide in 2019 ─ Global Health Estimates*. World Health Organization, Geneva. 2021. Available at: https://www.who.int/publications/i/item/9789240026643. Accessed 24 Jan 2023.

[15] India State-Level Disease Burden Initiative Suicide Collaborators (2018). Gender differentials and state variations in suicide deaths in India: the Global Burden of Disease Study 1990–2016. The Lancet Public Health.

[16] Knipe DW, Metcalfe C, Fernando R, Pearson M, Konradsen F, Eddleston M (2014). Suicide in Sri Lanka 1975–2012: age, period and cohort analysis of police and hospital data. BMC Public Health.

[17] Vijayakumar L, Daly C, Arafat Y, Arensman E (2020). Suicide prevention in the Southeast Asia region. Crisis: J Crisis Intervent Suicide Prevent.

[18] Vijayakumar L (2015). Suicide in women. Indian J Psychiatry.

[19] Knipe D, Metcalfe C, Hawton K, Pearson M, Dawson A, Jayamanne S (2019). Risk of suicide and repeat self-harm after hospital attendance for non-fatal self-harm in Sri Lanka: a cohort study. Lancet Psychiatry.

[20] Pushpakumara P, Thennakoon SUB, Rajapakse TN, Abeysinghe R, Dawson AH (2019). A prospective study of repetition of self-harm following deliberate self-poisoning in rural Sri Lanka. PLoS One.

[21] Pathare S, Shields-Zeeman L, Vijayakumar L, Pandit D, Nardokar R, Chatterjee S (2020). Evaluation of the SPIRIT integrated suicide prevention programme: study protocol for a cluster-randomised controlled trial in rural Gujarat, India. Trials.

[22] Karunarathne A, Gunnell D, Konradsen F, Eddleston M (2020). How many premature deaths from pesticide suicide have occurred since the agricultural green revolution?. Clin Toxicol (Phila).

[23] Dowler C. Thousands of tonnes of banned pesticides shipped to poorer countries from British and European factories; 2020 Sep 9. Available from: https://unearthed.greenpeace.org/2020/09/10/banned-pesticides-eu-export-poor-countries/. Accessed 24 Jan 2023.

[24] Gunnell D, Eddleston M, Phillips MR, Konradsen F (2007). The global distribution of fatal pesticide self-poisoning: Systematic review. BMC Public Health.

[25] Chowdhury FR, Dewan G, Verma VR, Knipe DW, Isha IT, Faiz MA (2018). Bans of WHO Class I Pesticides in Bangladesh-suicide prevention without hampering agricultural output. Int J Epidemiol.

[26] Knipe DW, Chang SS, Dawson A, Eddleston M, Konradsen F, Metcalfe C (2017). Suicide prevention through means restriction: Impact of the 2008–2011 pesticide restrictions on suicide in Sri Lanka. PLoS One..

[27] Phillips MR, Yang G, Zhang Y, Wang L, Ji H, Zhou M (2002). Risk factors for suicide in China: a national case-control psychological autopsy study. The Lancet.

[28] Phillips MR, Li X, Zhang Y (2002). Suicide rates in China, 1995–99. The Lancet.

[29] Liu S, Page A, Yin P, Astel-Burt T, Feng X, Liu Y (2015). Spatiotemporal variation and social determinants of suicide in China, 2006–2012: findings from a nationally representative mortality surveillance system. Psychol Med.

[30] Gunnell D, Fernando R, Hewagama M, Priyangika WDD, Konradsen F, Eddleston M (2007). The impact of pesticide regulations on suicide in Sri Lanka. Int J Epidemiol.

[31] Knipe DW, Gunnell D, Eddleston M (2017). Preventing deaths from pesticide self-poisoning—learning from Sri Lanka's success. Lancet Glob Health.

[32] Arya V, Page A, Gunnell D, Dandona R, Mannan H, Eddleston M (2019). Suicide by hanging is a priority for suicide prevention: method specific suicide in India (2001–2014). J Affect Disord.

[33] Ministry of Agriculture and Farmers Welfare (2020). The Gazette of India: Extraordinary; PART II—Section 3—Sub-section (ii).

[34] Arya V, Page A, Gunnell D, Armstrong G (2021). Changes in method specific suicide following a national pesticide ban in India (2011–2014). J Affect Disord.

[35] Ramesh P, Taylor PJ, McPhillips R, Raman R, Robinson C (2022). A scoping review of gender differences in suicide in India. Front Psych.

[36] Payne S. *How can gender equity be addressed through health systems?* World Health Organisation Regional Office for Europe, Copenhagen. 2009. Availabe from: https://apps.who.int/iris/handle/10665/107956. Accessed 24 Jan 2023.

[37] Dandona R, Kumar GA (2023). India's National Suicide Prevention Strategy: considerations to enhance desired outcomes. Lancet Psychiatry.

[38] Chang Q, Yip PSF, Chen Y-Y (2019). Gender inequality and suicide gender ratios in the world. J Affect Disord.

[39] Cai Z, Canetto SS, Chang Q, Yip PSF (2021). Women's suicide in low-, middle-, and high-income countries: Do laws discriminating against women matter?. Soc Sci Med.

[40] Parkar SR, Dawani V, Weiss MG (2008). Gender, suicide, and the sociocultural context of deliberate self-harm in an urban general hospital in Mumbai, India. Cult Med Psychiatry.

[41] Thompson N, Bhugra D (2000). Rates of deliberate self-harm in Asians: findings and models. Int Rev Psychiatry.

[42] Knipe D, Padmanathan P, Newton-Howes G, Chan LF, Kapur N (2022). Suicide and self-harm. The Lancet.

[43] Lee YY, Chrisholm D, Eddleston M, Gunnell D, Fleischmann A, Konradsen F (2021). The cost-effectiveness of banning highly hazardous pesticides to prevent suicides due to pesticide self-ingestion across 14 countries: an economic modelling study. Lancet Glob Health.

[44] Eddleston M, Gunnell D (2020). Preventing suicide through pesticide regulation. The Lancet Psychiatry.

[45] Gunnell D, Knipe D, Chang SS (2017). Prevention of suicide with regulations aimed at restricting access to highly hazardous pesticides: a systematic review of the international evidence. Lancet Glob Health.

